# Dataset allowing for the identification of three new synthetic cannabimimetics featuring a norbornyl methyl side chain by spectrometric and spectroscopic techniques

**DOI:** 10.1016/j.dib.2021.107628

**Published:** 2021-11-23

**Authors:** Benedikt Pulver, Jan Riedel, Torsten Schönberger, Michael Pütz, Jan Schäper, Natalie Kunert, Klaus Putzer, Gunter Hermann, Volker Auwärter, Folker Westphal

**Affiliations:** a Institute of Forensic Medicine, Forensic Toxicology, Medical Center – University of Freiburg, Faculty of Medicine, University of Freiburg, Albertstr. 9, Freiburg 79104, Germany; b Hermann Staudinger Graduate School, University of Freiburg, Hebelstr. 27, 79104 Freiburg, Germany; c State Bureau of Criminal Investigation Schleswig-Holstein, Forensic Science Institute, Mühlenweg 166, Kiel 24116, Germany; d Federal Criminal Police Office, Forensic Science Institute, Äppelallee 45, Wiesbaden 65203, Germany; e Bavarian State Criminal Police Office, Forensic Science Institute, Maillingerstr. 15, München 80636, Germany; f German Customs, Direction IX, Educational and Scientific Center, Lilienthalstraße 3, Markt Schwaben 85570, Germany; g Douane Laboratorium, Kingsfordweg 1, Amsterdam 1043 GN, the Netherland; h QoD Technologies GmbH, c/o Freie Universität Berlin, Altensteinstr. 40, 14195 Berlin, Germany

**Keywords:** Synthetic cannabinoid, NPS, Structure elucidation, Legislation, Norbornyl methyl side chain

## Abstract

Synthetic cannabimimetics (SC) are a diverse group of new psychoactive substances with varying potency and harm potential. New SCs appear on the drug market every year, and reliable and correct identification of these new derivatives independent from the matrix relies on the availability of verified spectra. Three new synthetic cannabimimetics featuring a norbornyl methyl side chain and varying core structure elements were identified in different seizures and forms. Cumyl-BC[2.2.1]HpMeGaClone and Cumyl-BC[2.2.1]HpMINACA were laced onto herbal blends, whereas Cumyl-BC[2.2.1]HpMICA was seized as a pure solid powder. The data collection process involves a comprehensive set of orthogonal analytical techniques allowing for the unambiguous identification of the respective endo- and exo-isomers.

Furthermore, the diversity of analytical techniques allows a greater number of laboratories working in the field of forensic chemistry to confidently identify the substances described in our original research article [1]. Structure elucidation and analytical characterisation were performed within the EU-project ADEBAR *plus* using gas chromatography-mass spectrometry (GC-MS), gas chromatography-solid state infrared spectroscopy (GC-sIR), as well as solid and neat IR spectroscopy, Raman spectroscopy, liquid chromatography-electrospray ionisation-mass spectrometry (LC-ESI-MS), and high resolution (HR)-LC-ESI-MS, and nuclear magnetic resonance (NMR) spectroscopy. The raw analytical data files are included in the Mendeley repository alongside the individual spectra in a universally importable format. The use of the universal JCAMP format for storage of the spectra facilitates database maintenance and enables seamless integration of the verified spectra. Thus, the dataset enables other researchers worldwide to identify these three new SCs confidently.

## Specifications Table

**Table:** 

Subject	Analytical Chemistry
Specific subject area	Analytical and Forensic Chemistry, Structural elucidation, and differentiation of new psychoactive substances, specifically synthetic cannabimimetics
Type of data	table graph figure
How the data were acquired	GC-EI-MS data was acquired using a Finnigan TSQ 8000 triple stage quadrupole mass spectrometer coupled to a Trace GC Ultra (Thermo Fisher, MA, US). Column: DB-1 (30 m × 0.32 mm i.d., 0.25 µm film thickness) (Agilent Technologies, CA, US). Sample introduction: CTC CombiPAL (CTC Analytics, Zwingen, Switzerland) autosampler. Software: Xcalibur 4.0. Additional information can be found in Table 1. Additional GC-EI-MS data on Cumyl-BC[2.2.1]HpMeGaClone was acquired using an ISQ single quadrupole mass spectrometer (Thermo Fisher) coupled to a Trace GC Ultra gas chromatograph (Thermo Fisher).Column: TG-5MS (30 m × 0.25 mm i.d., 0.25 µm film thickness) (Thermo Fisher). GC-sIR data was acquired using an Agilent GC 7890B (Waldbronn, Germany) with an Agilent G4567A probe sampler and a DiscovIR-GCTM (Spectra Analysis, Marlborough, MA, USA). Data were acquired and processed using GRAMS/AI Ver. 9.1 (Grams Spectroscopy Software Suite, Thermo Fischer Scientific, Dreieich, Germany) followed by OMNIC Software, Ver. 7.4.127 (Thermo Fisher). Additional method parameters can be found in Table 2. LC-ESI-MS/MS data was acquired using a Thermo Accela 1250 HPLC chromatograph coupled to a Thermo Velos Pro (linear ion trap) spectrometer with electrospray ionization; software: Xcalibur 4.0. Additional method parameters can be found in Table 3.
	ATR-FTIR spectra were acquired using a Nicolet iS20 FT-IR spectrometer with Smart iTX Diamond ATR. Software: OMNIC, Ver. 9.11.706 (Thermo Electron Corporation, Dreieich, Germany). ATR-FT-NIR data were acquired using Perkin Elmer 100 N FT-NIR spectrometer at ambient temperature through a glass vial. Raman data was averaged from ten consecutive scans acquired through a BAC151B Raman Video Microsampling System (B&W TEK, DE, USA). Additional details on the method parameters can be found in Table 5. NMR data were acquired using a AVANCE III HD 500 and 5 mm CryoProbe Prodigy BBO probe with z‐gradient (BRUKER BioSpin, Rheinstetten, DE). Additional acquisition parameters can be found in Table 4.
Data format	raw analysed
Description of data collection	SCs laced on herbal material were extracted using chloroform. The filtrate was used for GC-MS, LC-MS, GC-sIR and NMR analyses. Raman and IR data were acquired from the solid powder as received where possible.
Data source location	• Institution: State Bureau of Criminal Investigation Schleswig-Holstein, Forensic Science Institute • City/Town/Region: Kiel • Country: Germany
Data accessibility	Repository name: Mendeley Data Data identification number: 10.17632/m3dbvfss5v.1 Direct URL to data: https://data.mendeley.com/datasets/m3dbvfss5v/1
Related research article	[1] B. Pulver, J. Riedel, T. Schönberger, M. Pütz, J. Schäper, N. Kunert, K. Putzer, G. Hermann, V. Auwärter, F. Westphal, Comprehensive structural characterisation of the newly emerged synthetic cannabimimetics Cumyl-BC[2.2.1]HpMeGaClone, Cumyl-BC[2.2.1]HpMINACA, and Cumyl-BC[2.2.1]HpMICA featuring a norbornyl methyl side chain, Forensic Chemistry. https://doi.org/10.1016/j.forc.2021.100371.

## Value of the Data

• The confident identification of synthetic cannabimimetics requires verified analytical reference data from orthogonal techniques.
• The analytical dataset serves as verified analytical data for all laboratories working in the field of Forensic Chemistry tasked with the identification and differentiation of synthetic cannabimimetics
• The analytical data is supplied in readily importable formats enabling the rapid implementation into existing methods and workflows by other researchers around the world.

## 1 Data Description

The word document substance_profile.docx contains the overview on the three SCs including the IUPAC name, synonyms, molecular weight, retention indices derived from GC and LC analysis, and the sample ID assigned to the analysed seizures.

The analytical data files are stored in separate folders for each individual substance. The folder named 20_ADB-063 contains the analytical data on Cumyl-BC[2.2.1]HpMeGaClone. Folders named 20_ADB-085 and 21_ADB-004 contain the analytical data on Cumyl-BC[2.2.1]HpMINACA and Cumyl-BC[2.2.1]HpMICA, respectively.

### 1.1 Cumyl-BC[2.2.1]HpMeGaClone

20_ADB-063.raw contains the raw data of the GC-EI-MS analysis of the herbal blend extract of Cumyl-BC[2.2.1]HpMeGaClone on a DB-1 chromatographic column. The EI-MS spectra extracted from this raw dataset for the endo- and exo-isomer can be found in 20_ADB-063_GC-EI-MS_exo_isomer.jdx and 20_ADB-063_GC-EI-MS_endo_isomer.jdx, respectively. Additionally, GC-EI-MS analysis of the herbal blend extract on a DB-5 type chromatographic column was performed (Jul_437.raw).

The files 20_ADB-063_2nd_deposit.Absorbance.spc and 20_ADB-063_2nd_deposit.Multifile.cgm contain the raw analytical data on the GC-sIR analysis of the herbal blend extract. The dataset is the result of two consecutive injections that were deposited on top of each other following chromatographic separation. The resulting solid IR spectra of the individual endo- and exo-isomers can be found in 20_ADB-063_endo_isomer_(GC-sIR).jdx and 20_ADB-063_exo_isomer_(GC-sIR).jdx.

A neat IR spectrum of the herbal blend extract using chloroform as solvent was acquired and can be found in 20_ADB-063_neat_(CHCl3)_(mixture_of_isomers).jdx.

ESI-MS/MS spectra of Cumyl-BC[2.2.1]HpMeGaClone were acquired for the ion species [M+H]+ as well as the potassium and sodium adduct ions.
20_ADB-063_411-@17_10_LC-iontrap-MS.jdx contains the MS/MS spectrum of the [M+H]+ ion using a normalized collision energy of 17 without wideband excitation.
20_ADB-063_411-w@23_10_LC-iontrap-MS.jdx contains the MS/MS spectrum of the [M+H]+ ion using a normalized collision energy of 23 and wideband excitation.
20_ADB-063_sodium adduct_433-@26_10_LC-iontrap-MS.jdx contains the MS/MS spectrum of the [M+Na]+ ion using a normalized collision energy of 26 without wideband excitation.
20_ADB-063_sodium adduct_433-w@33_10_LC-iontrap-MS.jdx contains the MS/MS spectrum of the [M+Na]+ ion using a normalized collision energy of 33 and wideband excitation.
20_ADB-063_potassium_adduct_449-@30_10_LC-iontrap-MS.jdx contains the MS/MS spectrum of the [M+K]+ ion using a normalized collision energy of 30 without wideband excitation.
20_ADB-063_potassium adduct_449-w@40_10_LC-iontrap-MS.jdx contains the MS/MS spectrum of the [M+K]+ ion using a normalized collision energy of 40 and wideband excitation.

1D ^1^H and ^13^C NMR spectra acquired on the mixture of endo- and exo-isomers were exported from the raw data file after signal assignment and can be found in the repository under 1H Cumyl-BC[2.2.1]HpMeGaClone_raw.jdx and 13C Cumyl-BC[2.2.1]HpMeGaClone_raw.jdx, respectively. Additionally, extracted NMR spectra for the mixture of isomers were exported to files named 1H Cumyl-BC[2.2.1]HpMeGaClone_extracted.jdx and 13C Cumyl-BC[2.2.1]HpMeGaClone_extracted.jdx.

### 1.2 Cumyl-BC[2.2.1]HpMICA

21_ADB-004_dil.raw contains the raw data of the GC-EI-MS analysis of Cumyl-BC[2.2.1]HpMICA dissolved in chloroform. The EI-MS spectra extracted from this raw dataset for the endo- and exo-isomer can be found in 21_ADB-004_endo_isomer_GC-EI-MS.jdx and 21_ADB-004_exo_isomer_GC-EI-MS.jdx, respectively.

The files 21_ADB-004.Absorbance.spc and 21_ADB-004.Multifile.cgm contain the raw analytical data on the GC-sIR analysis. The resulting solid IR spectra of the individual endo- and exo-isomers can be found in 21_ADB-004_endo_isomer_(GC-sIR).jdx and 21_ADB-004_exo_isomer_(GC-sIR).jdx.

A neat IR spectrum of the substance using chloroform as solvent as acquired and can be found in 21_ADB-004 neat (CHCl3)_IR.jdx. The solid IR spectrum of the sample as received can be found in the file 21_ADB-004 solid.jdx. The solid Raman spectra at wavelengths of 785 nm and 1064 nm can be found in the files 21_ADB-004_mc_785_100_3000_10 and 21_ADB-004_mc_1064_100_65000_10, respectively. The file named 21_ADB-004_NIR.jdx contains the NIR spectrum of the sample as received.

ESI-MS/MS spectra of Cumyl-BC[2.2.1]HpMICA were acquired for the ion species [M+H]+, [M-H]- as well as the potassium adduct ion.
21_ADB-004_387@23_LC-iontrap-MS.jdx contains the MS/MS spectrum of the [M+H]+ ion using a normalized collision energy of 23 without wideband excitation.
21_ADB-004_387-w@29_LC-iontrap-MS.jdx contains the MS/MS spectrum of the [M+H]+ ion using a normalized collision energy of 29 and wideband excitation.
21_ADB-004_385@32_LC-iontrap-MS.jdx contains the MS/MS spectrum of the [M-H]- ion using a normalized collision energy of 32 without wideband excitation.
21_ADB-004_385-w@45_LC-iontrap-MS.jdx contains the MS/MS spectrum of the [M-H]- ion using a normalized collision energy of 45 and wideband excitation.
21_ADB-004_409-@34_LC-iontrap-MS.jdx contains the MS/MS spectrum of the [M+Na]+ ion using a normalized collision energy of 34 without wideband excitation.
21_ADB-004_409-w@52_LC-iontrap-MS.jdx contains the MS/MS spectrum of the [M+Na]+ ion using a normalized collision energy of 52 and wideband excitation.

1D ^1^H and ^13^C NMR spectra acquired on the mixture of endo- and exo-isomers were exported from the raw data file after signal assignment and can be found in the repository under 1H Cumyl-BC[2.2.1]HpMICA_raw.jdx and 13C Cumyl-BC[2.2.1]HpMICA_raw.jdx, respectively. Additionally, extracted NMR spectra for the mixture of isomers were exported to files named to files named 1H Cumyl-BC[2.2.1]HpMICA_extracted.jdx and 13C Cumyl-BC[2.2.1]HpMICA_extracted.jdx.

### 1.3 Cumyl-BC[2.2.1]HpMINACA

20_ADB-085.raw contains the raw data of the GC-EI-MS analysis of the herbal blend extract. The EI-MS spectra extracted from this raw dataset for the endo- and exo-isomer can be found in 20_ADB-085_GC-EI-MS_exo_isomer.jdx and 20_ADB-085_GC-EI-MS_endo_isomer.jdx, respectively.

The files 20_ADB-085.Absorbance.spc and 20_ADB-085.Multifile.cgm contain the raw analytical data on the GC-sIR analysis of the herbal blend extract. The resulting solid IR spectra of the individual endo- and exo-isomers can be found in 20_ADB-085_endo_isomer_(GC-sIR).jdx and 20_ADB-085_exo_isomer_(GC-sIR).jdx.

A neat IR spectrum of the herbal blend extract using chloroform as solvent as acquired and can be found in 20_ADB-085_neat_(CHCl3)_(mixture_of_isomers).jdx.
ESI-MS/MS spectra of Cumyl-BC[2.2.1]HpMINACA were acquired for the ion species [M+H]+ as well as the sodium adduct ion.
20_ADB-085_388@27_10_LC-iontrap-MS.jdx contains the MS/MS spectrum of the [M+H]+ ion using a normalized collision energy of 27 without wideband excitation.
20_ADB-085_388-w@31_10_LC-iontrap-MS.jdx contains the MS/MS spectrum of the [M+H]+ ion using a normalized collision energy of 31 and wideband excitation.
20_ADB-085_410@32_10_LC-iontrap-MS sodium adduct.jdx contains the MS/MS spectrum of the [M+Na]+ ion using a normalized collision energy of 32 without wideband excitation.
20_ADB-085_410-w@46_10_LC-iontrap-MS sodium adduct.jdx contains the MS/MS spectrum of the [M+Na]+ ion using a normalized collision energy of 46 and wideband excitation.

1D ^1^H and ^13^C NMR spectra acquired on the mixture of endo- and exo-isomers were exported from the raw data file after signal assignment and can be found in the repository under 1H Cumyl-BC[2.2.1]HpMINACA_raw.jdx and 13C Cumyl-BC[2.2.1]HpMINACA_raw.jdx, respectively. Additionally, extracted NMR spectra for the mixture of isomers were exported to files named 1H Cumyl-BC[2.2.1]HpMINACA_extracted.jdx and 13C Cumyl-BC[2.2.1]HpMINACA_extracted.jdx.

## 2 Experimental Design, Materials and Methods

### 2.1 GC-EI-MS analyses

Two of the three SCs (Cumyl-BC[2.2.1]HpMeGaClone and Cumyl-BC[2.2.1]HpMINACA) were not seized in the form of a pure powder but laced onto herbal plant material. The SCs were extracted from the herbal matrix using chloroform as solvent. The organic solvent and the herbal material were swirled only for a few seconds to decrease the amount of co-extracted constituents from the herbal matrix. The organic solvent was filtered through a 0.45 µm Whatman filter (GE Healthcare Life Sciences, BKM, UK) and kept for LC-MS, GC-sIR and NMR analyses. GC-MS data were acquired using a 1:10 dilution of the chloroform extract. GC-EI-MS parameters are outlined in Table 1.

**Table 1:** Method parameters of the GC-EI-MS analyses.

GC parameters:	injection volume: 1 µL, splitless; injector temperature: 280 °C; carrier gas: helium; flow rate: 1.2 mL/min. transfer line: 280 °C. temperature program: 80 °C, held for 1 min, followed by a ramp to 280 °C at 15 °C/min, held for 21 min. temperature program “310”: 80 °C, held for 2 min, followed by a ramp to 310 °C at 20 °C/min, held for 23 min.
MS parameters:	ionization mode: EI = 70 eV; emission current: 200 µA; ion source temperature: 175 °C; scan time: 1 s; scan range: m/z = 29 – 600.

The chromatographic separation on a DB-1 column was insufficient to facilitate the extraction of individual EI-MS spectra. Furthermore, the γ-carbolinone required the “310” temperature program to achieve elution from the column. The DB-5 column material was employed to achieve separation of the endo- and exo-isomers of Cumyl-BC[2.2.1]HpMeGaClone. For the GC-EI-MS analysis of Cumyl-BC[2.2.1]HpMICA, 2 mg of the compound were dissolved in 2 mL of chloroform. The extraction of spectra for the individual compounds and respective isomers as well as the determination of the Kovats retention index was done at the beginning of the peak resulting in a basepeak intensity that is below the saturation threshold of 108 for the instrument used. Background subtraction was performed at two points close to the analyte peak before exporting the resulting spectrum into the .jdx file format.

### 2.2 GC-sIR analyses

The sample preparation has been optimized to extract only minor fractions from the herbal blend to increase the concentration of the SC in the analysed solution as the analysis using GC-sIR requires a greater concentration of the analyte compared to GC-EI-MS. The rotating ZnSe disk is cleaned prior to the analysis of a new batch of samples to decrease the amount of ghost peaks caused by dust particles accumulating over time. Acetonitrile is used to wipe down the disk and residual, visible dust particles are removed using a stream of nitrogen just prior to reinserting the disk into the holding mechanism. Parameters of the GC-sIR analysis are outlined in Table 2.

**Table 2:** Method parameters of the GC-sIR analyses.

GC parameters:	injection: 1 µL, splitless mode; injection port temperature: 240 °C; carrier gas: helium; flow rate: 2.5 mL/min.
Chromatographic conditions:	fused silica capillary DB-1column (30 m × 0.32 mm i.d., 0.25 µm film thickness); oven temperature program: 80 °C for 2 min, ramped to 290 °C at 20 °C/min, and held at for 20 min; transfer line: 280 °C.
Infrared conditions:	oven temperature: 280 °C; restrictor temperature: 280 °C; disc temperature: −40 °C; dewar cap temperatures: 35 °C; vacuum: 0.2 mTorr; disc speed: 3 mm/min; spiral separation: 1 mm; wavelength resolution: 4 cm^−1^; IR range: 650-4000 cm^−1^; acquisition time: 0.6 s/file; 64 scans/spectrum.

The GC-sIR analysis of the herbal blend extract of Cumyl-BC[2.2.1]HpMeGaClone was deposited onto the cryogenically cooled twice to achieve a greater band intensity by overlaying two injections.

Following the acquisition of the GC-sIR data, the analyte peak is identified, and manual background subtraction is performed at one point next to the peak using GRAMS/AI Ver. 9.1 (Grams Spectroscopy Software Suite, Thermo Fisher). Next, multi-point baseline correction was performed over the complete scan range and the spectrum exported into the .jdx format [2].

### 2.3 LC-ESI-MS/MS analyses

MS/MS spectra are acquired for the SCs using the syringe pump to elucidate the collision induced fragmentation pathway and facilitate identification of the SCs via LC-MS/MS analysis workflows. The herbal blend extract was reconstituted in MeOH, diluted 1:10 injected into the ion source via a T-connector at a constant flow rate of 3-10 µL/min. 100% MeOH was supplied via the pump at a constant flow rate of 30 µL/min to aid in the stability of the ion spray at the interface. The collision gas was helium and the isolation width 1.2 (m/z).

The acquisition of the ESI-MS/MS collision spectra was performed with and without wideband excitation to account for different methods of operation by other forensic laboratories depending on the manufacturer used. In each case, the spectrum which shows the molecular ion with nearly 10% of the base peak intensity was chosen to retain the information on the [M+H]+ ion species in the MS spectrum.

The LC-MS/MS acquisition method includes a full scan and consecutive scans of product ions created from collision induced dissociation of the 7 and 5 most intense precursor ions in positive ion mode and negative ion mode, respectively, identified in the full scan. Additional method parameters can be found in Table 3.

**Table 3:** Method parameters of the LC-ESI-MS/MS analyses.

Column	Aqua C18 (3 µm, 150 × 3 mm, 125 Å)
Mobile phases	A: water with 0.0025% formic acid B: methanol with 0.0025% formic acid
Gradient	100% A for 3 min, then in 14 min to 98% B, held for 32 min, then to 100% A for 10 min
Flow rate	100 µL/min
Injection volume	1 µL
Column temperature	24 °C

The relative retention time (RRT) of the analyte using fluoresceine as internal standard was calculated using $\frac{RT_{analyte}}{RT_{fluoresceine}}$.

### 2.4 NMR analysis

For the measurement, the extracts were dissolved in deuterated acetone to which 0.1% TMS (trimethylsilane) was added as a reference for the spectra. Detailed parameters of the NMR data acquisition are detailed in Table 4.

**Table 4:** Parameters of the NMR acquisition of ^13^C and ^1^H data.

1D-^1^H	500 MHz, pulse program: zg, number of scans: 4, 90° pulse, spectral width: 17 ppm, transmitter offset: 5.5 ppm, time-domain: 128 k, spectrum size: 128 k, exponential multiplication with line broadening 0.2 Hz
1D-^13^C	125 MHz, pulse program: jmod (APT), number of scans: 512 or more, exponential multiplication with line broadening 1.0 Hz Peak assignments were corroborated by COSY, HSQC and HMBC.

After spectra acquisition, all spectra were imported into MNova, and the ^1^H and ^13^C spectra were saved in .jdx format (raw spectra). The imported spectra were referenced to TMS at 0 ppm for assignment. Phase and baseline corrections were also performed for the ^1^H and ^13^C spectra. Peak picking was performed using the GSD method in MNova (5 fitting cycles). For subsequent matching with other spectra, all previously fitted substance signals were converted to new spectra. These then contain neither noise, nor signals from minor components. The spectra were saved in jdx format (extracted spectra).

### 2.5 IR and Raman analysis

The solid powder of Cumyl-BC[2.2.1]HpMICA was analysed as received and no correction or modification of the spectrum was performed after the acquisition. Parameters of the Raman analyses are detailed in Table 5.

**Table 5:** Method parameters of the Raman analyses.

λ = 785 nm	BWS465-785S spectrometer scan range: 174 – 3200 cm^−1^; resolution: <4.5 cm^−1^ @ 912 nm (B&W TEK)
λ = 1064 nm	BWS485-1064S-05 spectrometer scan range: 170 – 2502 cm^−1^; resolution: ∼9.5 cm^−1^ @ 1296 nm (B&W TEK)

Solid IR and Raman data of Cumyl-BC[2.2.1]HpMeGaClone, Cumyl-BC[2.2.1]HpMINACA were not accessible as they were deposited onto a herbal blend. To facilitate identification of Cumyl-BC[2.2.1]HpMeGaClone, Cumyl-BC[2.2.1]HpMINACA via IR spectroscopy without a GC-sIR instrument, neat IR spectra using chloroform as solvent were acquired. The neat IR spectra are of the mixture of isomers as well as minor impurities from the herbal blend matrix material.

## CRediT authorship contribution statement

**Benedikt Pulver:** Writing – original draft, Investigation, Conceptualization. **Jan Riedel:** Investigation. **Torsten Schönberger:** Writing – review & editing. **Michael Pütz:** Resources, Writing – review & editing, Funding acquisition. **Jan Schäper:** Resources. **Natalie Kunert:** Resources. **Klaus Putzer:** Resources. **Gunter Hermann:** Investigation, Software. **Volker Auwärter:** Writing – review & editing. **Folker Westphal:** Writing – review & editing, Funding acquisition, Conceptualization.

## Declaration of Competing Interest

The authors declare that they have no known competing financial interests or personal relationships that could have appeared to influence the work reported in this paper.
